# Hepatitis E virus in Norway rats (*Rattus norvegicus*) captured around a pig farm

**DOI:** 10.1186/1756-0500-5-4

**Published:** 2012-01-05

**Authors:** Yuta Kanai, Satoshi Miyasaka, Sachiko Uyama, Sachiyo Kawami, Yuko Kato-Mori, Muneo Tsujikawa, Mikihiro Yunoki, Shoko Nishiyama, Kazuyoshi Ikuta, Katsuro Hagiwara

**Affiliations:** 1 School of Veterinary Medicine, Rakuno Gakuen University, Ebetsu, Hokkaido 069-8501, Japan; 2 Department of Virology, Research Institute for Microbial Diseases, Osaka University, Osaka 565-0871, Japan; 3 Infectious Pathogen Research Group, Osaka Research Laboratory, Research & Development Division, Benesis Corporation, Osaka 541-8505, Japan; 4 Present Address: Department of Infectious and Tropical Diseases, London School of Hygiene and Tropical Medicine, Keppel Street, London WC1E 7HT, UK

## Abstract

**Background:**

Hepatitis E virus (HEV) transmitted via the oral route through the consumption of contaminated water or uncooked or undercooked contaminated meat has been implicated in major outbreaks. Rats may play a critical role in HEV outbreaks, considering their negative effects on environmental hygiene and food sanitation. Although the serological evidence of HEV infection in wild rodents has been reported worldwide, the infectivity and propagation of HEV in wild rats remain unknown. To investigate if rats are a possible carrier of HEV, we studied wild Norway rats (*Rattus norvegicus*) that were caught near a pig farm, where HEV was prevalent among the pigs.

**Methods:**

We examined 56 Norway rats for HEV. RNA from internal organs was examined for RT-PCR and positive samples were sequenced. Positive tissue samples were incubated with A549 cell line to isolate HEV. Anti-HEV antibodies were detected by ELISA.

**Results:**

Sixteen rats were seropositive, and the HEV RNA was detected in 10 of the 56 rats. Sequencing of the partial *ORF1 *gene from 7 samples resulted in partially sequenced HEV, belonging to genotype 3, which was genetically identical to the HEV prevalent in the swine from the source farm. The infectious HEVs were isolated from the Norway rats by using the human A549 cell line.

**Conclusions:**

There was a relatively high prevalence (17.9%) of the HEV genome in wild Norway rats. The virus was mainly detected in the liver and spleen. The results indicate that these animals might be possible carrier of swine HEV in endemic regions. The HEV contamination risk due to rats needs to be examined in human habitats.

## Background

Hepatitis E virus (HEV) is a causative agent of viral hepatitis transmitted via the oral route in humans. The clinical symptoms of HEV infection vary from asymptomatic to acute fulminant hepatitis. In humans, pregnancy and underlying liver diseases are considered risk factors for severe cases of HEV [1,2] and high mortality rates have been reported among pregnant women [3-6]. Also, chronic hepatitis associated with HEV was recently reported in organ transplant recipients [7].

HEV is a non-enveloped single-stranded positive-sense RNA virus classified as the sole member of the genus *Hepevirus *in the family *Hepeviridae *[8]. The 7.2-kb genome of HEV is composed of 3 open reading frames (ORFs): ORF1 (a non-structural protein), ORF2 (a capsid protein), and ORF3 (accessory proteins associated with virion cellular protein kinase activity and virion release) [9-12].

HEVs associated with human hepatitis are classified into 4 genotypes [13]. Genotypes 1 and 2 of HEVs cause waterborne diseases, these genotypes are endemic in developing countries and cause outbreaks involving large populations [14,15]; meanwhile, genotypes 3 and 4 are generally considered zoonotic HEVs. Molecular analysis of the virus in patients and contaminated food provide evidence for direct food-borne transmission of the virus [16-18]. Recently, HEVs were detected in rabbits in China and rats in Germany; however, their infectivity to humans remains undetermined [19,20].

Epidemiological studies suggest that pigs are an important virus source of human HEV infections [21-25]. Many studies have shown that HEV infection occurs in many other animals in addition to pigs as evidenced by the detection of the HEV RNA or HEV-specific antibodies [19,26-28]. Although the prevalence of HEV-specific antibodies in wild rodents is well documented [28-31], there is only a single report of HEV isolated from rats in Europe [20]. Besides natural infection, a few cases of successful experimental infections of HEV genotype 1 to Wistar rats [32], HEV genotype 3 to Mongolian gerbils [33], and HEV genotype 4 to nude mice [34] have been reported. Although the transmission of the virus from wild rodents to domestic animals (e.g., pigs) is possible, the extent of this risk remains unknown [35]. To investigate whether rodents can serve as reservoirs of porcine HEV, we examined HEV infection in wild rats caught around a pig farm where HEV infection was prevalent. To determine whether wild rats are reservoirs of swine HEV, we performed viral genome detection by reverse transcription-polymerase chain reaction (RT-PCR), serological examination, and virus isolation in wild rats.

## Methods

### Animals

Norway rats (*Rattus norvegicus*) were caught, using commercial snap traps at 6 different locations, around a pig farm where HEV genotype 3 was prevalent among pigs (Hokkaido, Japan) [36]. The farm consists of three buildings. The capture locations surrounded 3 different buildings where the grow-finishing pigs (about 120 total head counts) were reared. Our previous study revealed that all the pigs were infected with HEV genotype 3. These studies were performed in accordance with the guidelines for the capture, handling, and care of mammals of the Mammalogical Society of Japan. All animal experiments were approved by the Rakuno Gakuen University Ethical Committee for Animal Experiment Regulation, Hokkaido, Japan (approved #VH21C10).

The power analysis used to calculate the number of animals required for the study was performed on the basis of the 95% confidence interval shown on the Raosoft^® ^website (http://www.raosoft.com/samplesize.html). Because the prevalence rate of HEV infection in wild rodents was estimated to be approximately 10% [28-31], the values required for calculating the sample size were set as follows: infection rate, 10%; margin of error that determines the range of the 95% confidence interval, 5%; and confidence level that refers to the likelihood of 95% confident interval, 90%. The power analysis indicated the appropriate sample size to be 97 to obtain 10% HEV infection in rats with a 5% margin of error. Since we found a sufficient number of HEV infections in rats when 56 animals were examined, no further capturing was performed.

### Sampling and RNA extraction

The liver, spleen, intestines, and blood were collected from 56 wild rats to determine the presence of HEV RNA. Tissue and serum samples were collected from each rat, and the samples were stored at -80°C until analysis. Blood was collected from the hearts of dead wild rats by using filter paper (Toyo Roshi; Advantec, Tokyo, Japan) according to the manufacturer's instructions. The filter paper was dissolved in 1 mL of phosphate-buffered saline (PBS) and subsequently diluted (1:25) to make serum samples. During tissue collection from wild rats, each dissection instrument was sterilized to avoid contaminating tissues with HEV RNA. Tissue samples (100 mg) were homogenized using zirconia beads with a TissueLyser (Qiagen GmbH, Hilden, Germany). Viral RNA was extracted from 100 mg of the tissue sample by using 1 mL of TRIzol reagent (Life Technologies Corp., Carlsbad, CA) according to the manufacturer's instructions. The final elution was carried out using 50 μL of RNase-free H_2_O. The RNA extracted from HEV-infected (genotype 3) swine livers was used as a positive control.

### ELISA

Anti-HEV antibodies were detected by ELISA with a commercial kit (Viragent HEV-Ab kit; Cosmic Corporation, Tokyo, Japan) that used a truncated recombinant HEV ORF2 protein expressed in silkworm pupae [37] according to the manufacturer's instructions. For the positive control serum, 3 Wistar rats were subcutaneously immunized with ORF2 antigen (100 μg/rat) 3 times every 2 weeks. The recombinant ORF2 protein was produced as a fusion protein with glutathione-*S-*transferase (GST) from the plasmid, pGEX-AC2.1, which encodes the ORF2 antigen (genotype 3). TALON Metal Affinity Resin (Clontech Inc., Palo Alto, CA) was used to purify the recombinant ORF2 protein. The sera from 5 intact Wistar rats were used as negative controls. The serum samples were diluted in buffer (1:100) and were incubated for 1 h at room temperature. Because the ELISA kit was developed for detecting human antibodies, an HRP-conjugated anti-rat IgG antibody (Zymed Inc., South San Francisco, CA) was used as the secondary antibody. After the secondary antibody reactions, 50 μL of TMB (3,3',5,5'-tetramethylbenzidine) (Kirkegaard & Perry Laboratories Inc., Baltimore, MD) was added; after 30 min incubation at room temperature, 50 μL of 2 M sulfuric acid was added to stop the reaction. The optical density at 450 nm (OD_450_) was measured. The cutoff value for IgG ELISA was calculated as the mean OD + 3 SDs of 5 uninfected Wistar rats (cutoff: OD 0.3).

### RT-PCR and sequence analysis

Initially, the HEV RNA of the 5' terminal region of ORF1 was detected by semi-nested RT-PCR [36] with the sense primer HE61 (5'-CACRTATGTGGTCGAYGCCATGGAG-3'; R = A or G, Y = C or T) and the antisense primer HE51 (5'-GCCKRACYACCACAGCATTCG-3'; K = G or T) for reverse transcription (RT) and first round of PCR and the internal sense primer HE50 (5'-AAGGCTCCTGGCRTYACWAC-3'; W = A or T) for the second round of PCR to confirm the first round of PCR products. Reverse transcription and first-round amplification were carried out using the OneStep RT-PCR Kit (Qiagen). In each reaction, 5-μL aliquots of viral RNA solution were used. The reactions were performed in an Eppendorf Mastercycler (Eppendorf, Hamburg, Germany) under the following conditions: RT at 50°C for 30 min, denaturation at 95°C for 15 min; 45 cycles of denaturation at 95°C for 15 s each, annealing at 55°C for 30 s, and elongation at 72°C for 30 s; and final extension at 72°C for 7 min. After the first round of PCR, 1-μL PCR product was amplified under the following conditions: 20 cycles of denaturation at 95°C for 15 s each, annealing at 60°C for 30 s, and elongation at 72°C for 15 s, followed by a final incubation at 72°C for 7 min. The amplified second-round PCR products were confirmed by 2% gel electrophoresis. The expected amplicon sizes of ORF1 were 125 bp and 85 bp in the first- and second-round PCRs, respectively. The RT-PCR-positive samples were also confirmed by semi-nested RT-PCR for the ORF 2 region by using the sense primer HE169 (5'-GAGGAGGAGGCTACTTCCG-3') and the antisense primer HE 171 (5'-CAGCCGACGAAATCAATTCTGTCG-3') for the RT and first-round PCR, and for the semi-nested PCR primer HE170 (5'-GTAATGCTTTGCATTCACGGCTCC-3'). The expected amplicon sizes of ORF2 for the first- and second-round PCRs were 373 bp and 349 bp, respectively. A 349-bp PCR amplicon was determined to be specific to HEV.

The PCR products from 7 positive samples (3 spleens and 4 intestines from 6 rats captured at 6 different locations) were excised from the gel, purified using QIAquick Gel Extraction Kit (Qiagen) and sequenced to confirm the specificity of the RT-PCR reaction. The purified products were cloned into a plasmid (pTA2, Cat: TAK-101; Toyobo Co. Ltd., Osaka, Japan) and sequenced using the M13 forward and reverse primers. The genomic sequences of the 5' terminal region of HEV ORF1 were compared using Bio Edit (version 7.0.9.0) (http://www.mbio.ncsu.edu/BioEdit/bioedit.html).

### Virus isolation from wild Norway rats

To isolate infectious rat HEVs, both the spleens and intestines of 3 of the HEV-positive wild rats (#38, #49, #50) were homogenized, filtered through a 0.22-μm Millipore filter (Millipore, MA), and inoculated into A549 cells (human alveolar basal epithelial cells), which are sensitive to HEV infection [38-40]. After adsorption for 60 min, the cells were washed with PBS and cultured in DMEM (Sigma-Aldrich Corp., St. Louis, MO) containing 2% fetal bovine serum and insulin-transferrin-selenium-X supplement (Cat. 51500-056; Life Technologies Corp., Carlsbad, CA) at 37°C in a 5% CO_2 _incubator [40]. The cells were passaged 3 days post-infection (dpi) and cultured in the same medium conditions. At 7 dpi, viral RNA was extracted from culture supernatants and cells by using a QIAamp Viral RNA Mini Kit (Qiagen). HEV RNA was examined by semi-nested RT-PCR for the ORF1 region as described above.

### Immunofluorescence assay

HEV antigen was detected by immunofluorescence assay to confirm the infectivity of rat HEV. Virus-infected cells (7 dpi) fixed with acetone-methanol for 5 min were washed with PBS and reacted with pig anti-HEV polyclonal antibody purified from pigs infected with HEV genotype 3 [41]. After incubation for 60 min at room temperature, the cells were washed with PBS containing 0.1% Tween 20 and reacted with FITC-rabbit anti-porcine IgG (Zymed) for 60 min at room temperature. After washing with the buffer, stained cells were examined under a Zeiss Pascal confocal microscope (LSM 5; Carl Zeiss AG, Oberkochen, Germany).

## Results

We captured 56 wild Norway rats at 6 different locations around a pig farm where HEV was detected among the pigs. Anti-HEV-specific antibodies in wild rats were examined by ELISA; 16 of the 56 (28.6%) rats were seropositive. HEV RNA ORF1 was detected in 10 of the 56 rats by semi-nested RT-PCR (17.9%): 5 in the spleen, 5 in the intestines, and 1 (rat ID #49) in both. HEV RNA at the ORF2 region was also detected in all of the HEV-RNA (ORF1)-positive samples. Six of the 10 RT-PCR-positive rats were seropositive in ELISA (Table 1). To confirm the specificity of the RT-PCR reaction, PCR products were purified and sequenced. These PCR products were obtained from tissues samples of rats captured at different locations, 3 spleens (#36, #38, and #49) and 4 intestines (#27, #43, #49, and #50). The nucleotide sequences of the partial ORF1 region of rat and swine HEV were closely related (swJB-M8, DDBJ: AB481228). Swine HEV genotype 3 (swJB-M8) was isolated from pigs in the same farm where rat HEV was isolated [42]. All 7 nucleotide sequences from the rats were similar and exhibited the greatest similarity to HEV genotype 3 (swJB-M8, 95.2-100%) followed by genotype 1 (81.8-88.6%), genotype 4 (77.2-84.1%), rabbit HEV-like virus (79.5%), genotype 2 (77.3%), avian HEV-like virus (68.2-70.5%), and rat HEV-like virus (56.8%).

**Table 1:** Summary of HEV RNA-positive Norway rats

Rat ID	Sex	Length (cm)	HEV-RNA			ELISA OD_450_	Virus Isolation
					
			Liver	Spleen	Intestine		
11*	ND	ND	**-**	**-**	**+**	0.10	NT

27	Male	15	**-**	**-**	**+**	0.33	NT

36	Male	15	**-**	**+**	**-**	0.17	NT

38	Female	20	**-**	**+**	**-**	0.38	+

40	Male	20	**-**	**+**	**-**	0.30	NT

43	Female	15	**-**	**-**	**+**	0.30	NT

49	Male	19	**-**	**+**	**+**	0.39	+**

50	Male	18	**-**	**-**	**+**	0.68	+

51	Male	15	**-**	**-**	**+**	0.60	NT

53	Male	19	**-**	**+**	**-**	0.49	NT

* ND: the rat could not be measured because of sample damage

** HEV was isolated from both the spleen and intestines. NT: not examined

**Figure 1:**
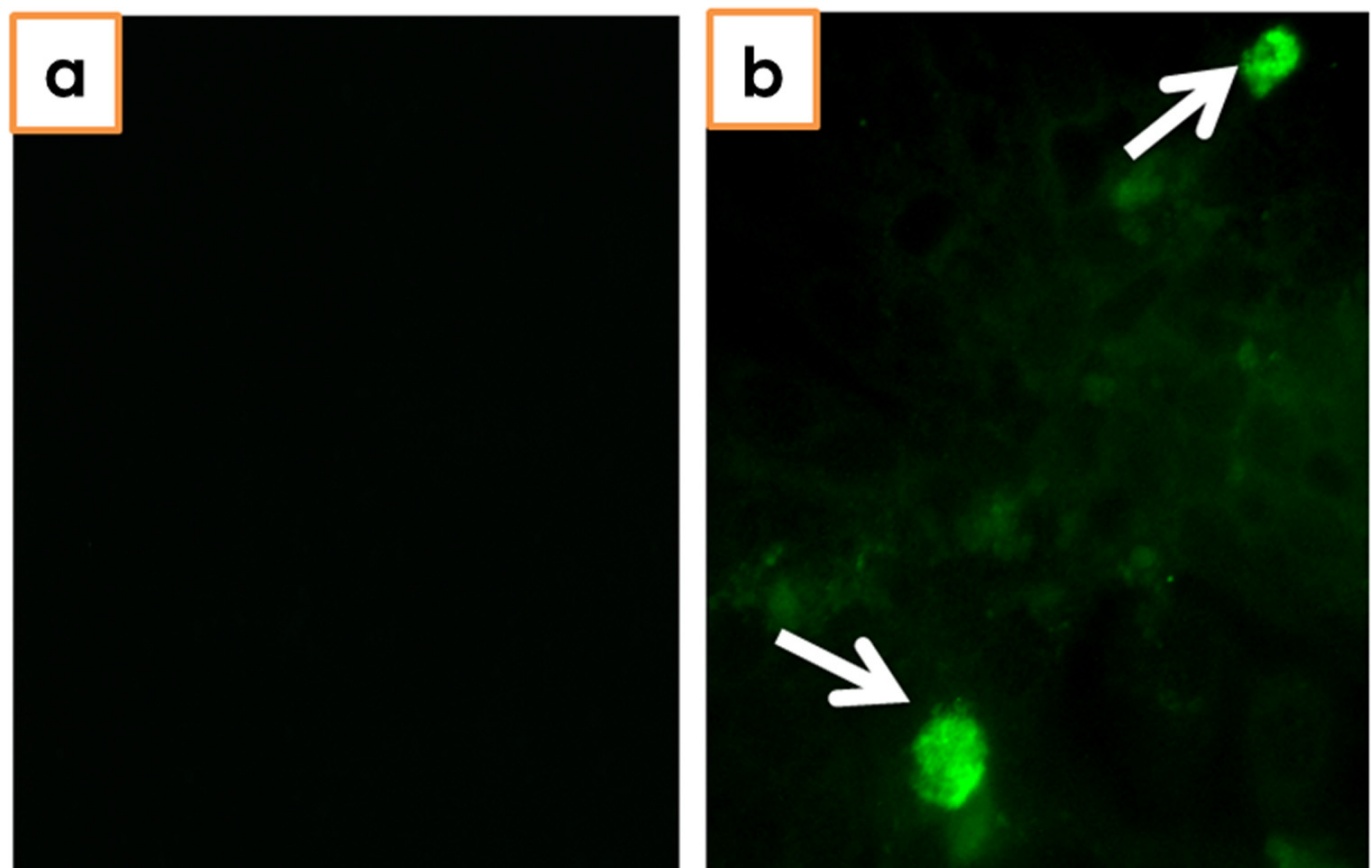
**Detection of HEV-infected cells by immunofluorescence assay**. **a **A549 cells of uninfected negative controls. **b **HEV-specific antigens were detected in A549 cells infected with splenic homogenate from rat #38.

To examine if the wild rats were carrying infectious HEV, we infected A549 cells with both splenic and intestinal homogenates from 3 HEV-positive rats (#38, #49, and #50). Although no apparent cytopathic effects were observed in A549 cells after inoculation, HEV RNA was detected in all samples by RT-PCR from both the supernatants and cells at 7 dpi. The presence of HEV antigen in the A549 cells was confirmed by immunofluorescence assay by using pig anti-HEV polyclonal antibodies (Table 1 and Figure 1).

## Discussion

More than 40% of all rats from different regions of the United States are reported to be seropositive for HEV [43]. Rats may be infected with HEV after coming in contact with infected domestic animals or contaminated sewage. Epidemiological studies indicate that HEV infection in pigs is ubiquitous and that most pigs older than 3 months are seropositive [44-46]. Although HEV shedding in feces is observed in pigs of all ages, it is more frequently observed in 2- to 4-month-old pigs than in slaughter-age (6-month-old) or adult pigs [23,47-50]. In this study, we found several rat nests around the farm; the rats also often moved through the pig pens. Therefore, rats can easily infect HEV by coming in contact with contaminants.

There are many studies on the presence of an anti-HEV-specific antibody in wild rodents, including the reports on the detection of an HEV-like virus from Norway rats in Germany [20,51]. In this study, the partial sequence of HEV from rats was closely related to the swine HEV genotype 3 (swJB-M8). HEV RNA was detected not only in the intestines but also in the spleens of these rats, suggesting that HEV infects and replicates in wild rats. In this study, HEV RNA was not detected in liver samples. It is possible that RNA was rapidly degraded postmortem in this tissue. In addition, it is possible that only a small quantity of HEV was present in the samples.

Norway rats live, to great extent, in close association with humans. Although the dynamics of HEV in wild rats is unclear, there is a concern of the possibility of HEV spreading from rats to other species, including humans. Pigs are considered as the most important carrier of HEV because of the high prevalence of HEV among domesticated pig populations [36,44,45,48,50]. The fact that infectious HEV is found in pig manure [52] also emphasizes the importance of controlling HEV infection among pigs.

The risk factors for HEV infection are related to poor sanitation in endemic regions as well as HEV shedding in feces and subsequent water or food contamination in human habitats. In Asia, including Japan, pig farms and human habitats are adjacent; therefore, wild rats frequently enter and leave areas of human habitation. In this study, rats carried swine HEV and the HEV from rats was infectious to human A549 cells. The transmission of HEV from pigs to humans has been demonstrated via the consumption of undercooked or uncooked meat. Therefore, high hygienic standards in human habitats and in pig farms are an important issue to prevent the transmission of HEV, and rodent control may be a critical aspect to address the issue.

## Conclusions

In this study, 10 of the 56 (17.9%) rats captured around a pig farm were positive for HEV genotype 3, and they were carrying infectious viruses. The contamination risk of HEV via rats needs to be studied in further detail.

## Competing interests

The authors declare that they have no competing interests.

## Authors' contributions

YK, SM, SU, SK, YKM, MT, SN, and KH performed the molecular biology and serological examination of the rats. SM, MY, KI, and KH isolated the viruses and helped draft the manuscript. MY, KI, and KH conceived the study, participated in its design and coordination, and drafted the manuscript. All authors have read and approved the final manuscript.
